# Helium Nanodroplet Infrared Action Spectroscopy of the Proton-Bound Dimer of Hydrogen Sulfate and Formate: Examining Nuclear Quantum Effects

**DOI:** 10.1021/acs.jpca.1c05705

**Published:** 2021-10-15

**Authors:** Daniel A. Thomas, Martín Taccone, Katja Ober, Eike Mucha, Gerard Meijer, Gert von Helden

**Affiliations:** Fritz-Haber-Institut der Max-Planck-Gesellschaft, Faradayweg 4−6, 14195 Berlin, Germany

## Abstract

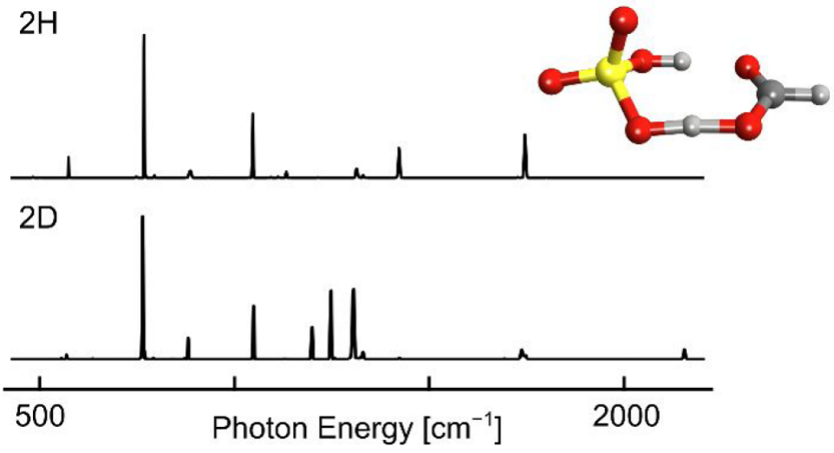

The proton-bound dimer of hydrogen sulfate and formate is an archetypal structure for ionic hydrogen-bonding complexes that contribute to biogenic aerosol nucleation. Of central importance for the structure and properties of this complex is the location of the bridging proton connecting the two conjugate base moieties. The potential energy surface for bridging proton translocation features two local minima, with the proton localized at either the formate or hydrogen sulfate moiety. However, electronic structure methods reveal a shallow potential energy surface governing proton translocation, with a barrier on the order of the zero-point energy. This shallow potential complicates structural assignment and necessitates a consideration of nuclear quantum effects. In this work, we probe the structure of this complex and its isotopologues, utilizing infrared (IR) action spectroscopy of ions captured in helium nanodroplets. The IR spectra indicate a structure in which a proton is shared between the hydrogen sulfate and formate moieties, HSO_4_^–^···H^+^···^–^OOCH. However, because of the nuclear quantum effects and vibrational anharmonicities associated with the shallow potential for proton translocation, the extent of proton displacement from the formate moiety remains unclear, requiring further experiments or more advanced theoretical treatments for additional insight.

## Introduction

The ionic hydrogen bond is a prominent structural motif found in diverse chemical systems. The mechanical and functional significance of this linkage arises from its unique properties, including strong interaction energies on the order of 20–150 kJ mol^–1^ and the capability to participate in proton transfer reactions.^1−3^ Of particular interest is the complex formed when two anionic Brønsted conjugate bases are bridged by a single proton, yielding a singly charged anionic moiety, here denoted as AHA^–^. Such structures are found, for example, in proteins,^3−6^ aerosol prenucleation clusters,^7−13^ and ionic liquids.^14−16^ The precise location of the bridging proton in these complexes—specifically, whether the proton is shared (A^–^···H^+^···A^–^) or localized on a single residue (AH···A^–^)—has been the subject of extensive research in systems ranging from deprotonated water clusters to proteins.^2,3,5,17−20^ These inquiries are motivated by the consequences of proton location for both overall structure and molecular properties such as effective p*K*_a_.^2,21^

Infrared (IR) action spectroscopy of isolated molecular ions and ionic clusters has proven to be an effective technique for the experimental characterization of AHA^–^ systems.^12,22−25^ This methodology combines the selectivity afforded by mass spectrometry with the detailed structural insight attainable with IR spectroscopy, enabling a comprehensive analysis of the system of interest. Cryogenic ion IR spectroscopy, in which ions are buffer-gas cooled to temperatures of 10–100 K prior to spectroscopic interrogation, has proven particularly effective.^12,22^ This technique has been applied to the study of deprotonated water clusters,^17,19^ model carboxylate proton-bound dimers,^26−28^ and putative aerosol nucleation clusters.^12,13^ The structure of AHA^–^ systems has also been investigated utilizing photoelectron spectroscopy^9−11,29^ and room-temperature IR action spectroscopy.^24,25^

Because of the low mass of the proton and the small energetic barriers (often <10 kJ mol^–1^) governing proton translocation, it is essential to treat these proton-bound dimer complexes as quantum systems and account for nuclear quantum effects (NQEs) when assessing molecular structure and properties.^21,30−33^ In cases where the proton translocation barrier is sufficient to yield localization at a single acidic site (i.e., to yield an acid–conjugate base complex of the form AH···A^–^), NQEs typically yield a slight increase in the effective A–H bond length and a corresponding increase in acidity and hydrogen bond strength.^31,34^ For systems with low barriers, the vibrational zero-point energy can exceed the barrier height,^17,33,35^ and the inclusion of NQEs is therefore indispensable for an accurate description of proton location and thus molecular properties. This phenomenon is observed prominently, for example, in the HO^–^···H^+^···^–^OH complex, where the zero-point energy yields a fully symmetric structure featuring an equally shared proton.^17,18,20,35−38^

**Scheme 1:**
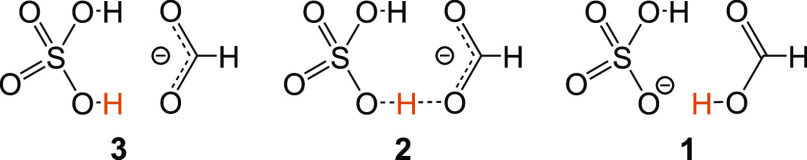
Skeletal-Formula Depiction of Structures of the Proton-Bound Heterodimer of Hydrogen Sulfate and Formate^9^

This work is concerned with the structure and properties of the proton-bound heterodimer of hydrogen sulfate and formate, [HSO_4_^–^ + H^+^ + HCOO^–^]^−^, a simple constituent of a class of organic–inorganic acid complexes that function in the nucleation of biogenic aerosol.^9−11^ As illustrated in Scheme 1, this complex is particularly intriguing because it features two hydrogen-bonding motifs between conjugate base moieties. A previous study on this system identified two low-energy structures: one corresponding to a sulfuric acid–formate cluster, H_2_SO_4_(HCOO^–^) (**3**, Scheme 1), and one corresponding to a hydrogen sulfate–formic acid cluster, HSO_4_^–^(HCOOH) (**1**, Scheme 1).^9^ These two local minima were found to be connected by a low-lying transition state featuring a shared proton (**2**, Scheme 1). The temperature dependence of the negative-ion photoelectron spectra was attributed to the shifts in the equilibrium population of these two structures resulting from the difference in free energy.

Herein, we study this complex and its isotopologues by IR action spectroscopy of ions captured in helium nanodroplets. The helium nanodroplet environment provides an ideal matrix for IR spectroscopy, yielding an equilibrium temperature of ca. 0.4 K while inducing minimal spectral perturbation.^27,39,40^ The obtained IR spectra are consistent with the presence of a single structure featuring a proton shared between the hydrogen sulfate and formate moieties, HSO_4_^–^···H^+^···^–^OOCH. The shared nature of the bridging proton is attributed to zero-point energy effects that yield a net displacement of the proton from the minimum on the potential energy surface (structure **1**). The magnitude of the barrier governing proton translocation is found to be highly sensitive to the level of theory employed in electronic structure calculations, yielding a significant challenge for the assessment of bridging proton location.

## Methods

### Experimental Methods

Helium nanodroplet infrared action spectroscopy experiments were performed utilizing custom instrumentation described in previous publications.^34,41−43^ The experimental apparatus comprises a modified quadrupole time-of-flight mass spectrometer with atmospheric pressure interface (Micromass Q-TOF Ultima, Waters Corporation, Milford, MA) coupled to custom components for helium nanodroplet generation, ion capture, droplet irradiation, and ion detection.

In this work, ions were generated from aqueous solutions utilizing nanoelectrospray ionization (nESI) from Pd/Pt-coated pulled glass capillaries fabricated in-house. To prepare electrospray solutions, formic acid (purity >98%), sulfuric acid (96%), and HPLC-grade water were purchased from Merck KGaA (Darmstadt, Germany). Deuterium oxide and isotopically labeled sodium formate (Na^18^O_2_CH) were purchased from Cambridge Isotope Laboratories (Tewksbury, MA). For spectroscopy of unsubstituted and deuterated analytes, ions were generated from a solution of 0.5% sulfuric acid, 5% formic acid, 47% water, and 47% methanol (v/v). A cone gas of D_2_O-saturated nitrogen was utilized to achieve deuterium substitution of exchangeable hydrogens. For spectroscopy of the complex containing ^18^O-substituted formate, a solution containing 100 mM labeled sodium formate (Na^18^O_2_CH) and 20 mM sulfuric acid in a 1:1 (v/v) methanol/water solution was used for nESI.

Ions generated by nESI and transferred to vacuum through the atmospheric pressure interface were selected utilizing a quadrupole mass filter, deflected 90° by a DC ion bender, and transferred to a 30-cm-long, helium-buffer-gas ion trap held at room temperature (ca. 298 K). For each spectroscopic data point, the ion trap was loaded for 2.0 s and subsequently evacuated of buffer gas over 1.5 s. The stored ions were then captured by traversing helium nanodroplets containing on average 20 000 He atoms,^43^ generated by a pulsed Even–Lavie valve.^44^ The nanodroplet-entrained ions escaped the shallow axial potential of the ion trap and traveled to a ring-electrode ion guide, where they were irradiated with IR photons generated by the Fritz Haber Institute free-electron laser (FHI FEL).^45^ The FEL macropulse duration was ca. 10 μs, with a micropulse duration of ca. 5 ps at a repetition rate of 1 GHz. The laser line width of the FEL was approximately 0.4% (full width at half-maximum) of the incident photon frequency. Resonant absorption of IR photons by the entrained ions resulted in the evaporation of helium atoms through energy redistribution to the nanodroplet, and the sequential absorption of multiple photons yielded bare ions, which were confined radially in the ion guide by a radio frequency potential. Following the FEL macropulse, these bare ions were pulsed into an off-axis time-of-flight detector.^42^

The cycle of nanodroplet generation, irradiation, and time-of-flight detection was repeated 25 times at a frequency of 10 Hz for each photon energy, and the integrated intensity of the averaged time-of-flight signal was utilized to generate an IR action spectrum. A photon-energy step size of 2 cm^–1^ was used for measurements above 1000 cm^–1^, and a step size of 1 cm^–1^ was used below 1000 cm^–1^. The ion trap filling cycle was repeated for each scan step.

To achieve optimal signal intensity at the time-of-flight detector, the photon fluence in the irradiation region was altered by adjusting the focal length of an adaptive IR mirror (A90/70, Kugler GmbH, Salem, Germany) in each scan region. The intensity of the spectra was subjected to a first-order power correction by dividing by the measured FEL macropulse energy, and the relative intensity of different scan regions was approximated by calibrating to the intensity of spectral lines measured in overlapping scans. Each presented spectrum represents an interpolated average of two to three scans.

### Electronic Structure Methods

Electronic structure calculations were carried out using the Gaussian 16 software package.^46^ Local minima of the [HSO_4_^–^ + H^+^ + HCOO^–^]^−^ complex previously identified by Hou, Wang, and Valiev^9^ were optimized at the B3LYP-D3BJ/aug-cc-pVTZ,^47−50^ MP2/aug-cc-pVTZ,^51^ and DSDPBEP86/aug-cc-pVTZ^52,53^ levels of theory using standard optimization settings within Gaussian 16. Additionally, energies of structures optimized at the MP2/aug-cc-pVTZ level of theory were computed using a complete basis set (CBS) extrapolation scheme^54^ with the aug-cc-pV*n*Z (*n* = 3, 4, 5) basis sets^50,55,56^ and a correction for the difference in correlation energy between CCSD(T)^57−60^ and MP2 with the aug-cc-pVTZ basis set.^61^ The transition state structure was initially identified utilizing a quadratic synchronous transit (QST) approach^62,63^ at the B3LYP-D3BJ/aug-cc-pVTZ level of theory and subsequently optimized with the Berny algorithm^64^ employing GEDIIS,^65^ as implemented in Gaussian with the Opt = TS keyword. Transition states were optimized at all levels of theory employed for the local minima.

A two-dimensional relaxed coordinate scan, in which the lengths of the SO–H bonds were fixed while all other atom positions were optimized, was performed at the DSDPBEP86/aug-cc-pVTZ level of theory using standard optimization settings within Gaussian 16. The step size for each SO–H bond scan was set to 0.05 Å. Following optimization, single-point energy calculations were carried out for each scan point at the CCSD(T)/aug-cc-pVTZ level of theory.

IR frequencies of [HSO_4_^–^ + H^+^ + HCOO^–^]^−^ and [DSO_4_^–^ + D^+^ + HCOO^–^]^−^ were calculated within the harmonic approximation using analytic second derivatives at the DSDPBEP86/aug-cc-pVTZ level of theory. For comparison to experiment, frequencies were scaled by a factor of 0.985 and convoluted with Gaussian functions of width 0.4% (full width at half-maximum) of the vibrational frequency.

## Results and Discussion

**Figure 1:**
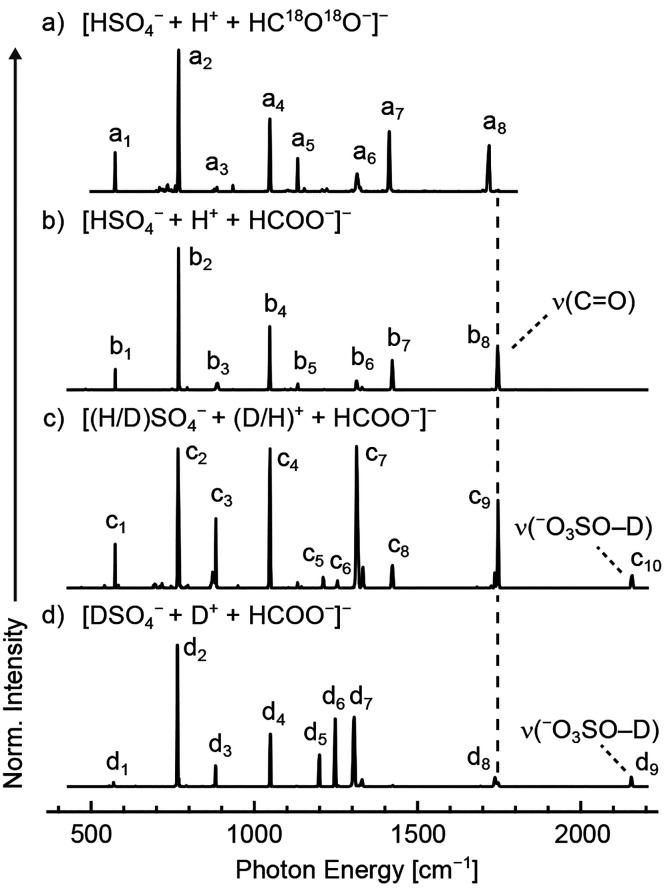
Experimental IR action spectrum of [HSO_4_^–^ + H^+^ + HCOO^–^]^−^ and its isotopologues captured in helium nanodroplets. A shift of the C=O stretching band is observed upon ^18^O substitution at the formate residue (line b_8_ vs line a_8_, panels b and a), and a band corresponding to the O–D stretching mode of the hydrogen sulfate moiety appears upon deuterium substitution of exchangeable hydrogens (lines c_10_ and d_9_, panels c and d). Upon deuterium substitution of both exchangeable hydrogens (panel d), direct corollaries of lines b_5_ and b_7_ are not readily identified, and new lines d_5_ and d_6_ appear.

Figure 1 shows the IR action spectra of the [HSO_4_^–^ + H^+^ + HCOO^–^]^−^ complex and its isotopologues obtained by action spectroscopy of ions captured in helium nanodroplets.^39,43,66^ Similar to previous results for analogous systems,^27,34,39^ the spectral lines are well resolved, with the spectral line width generally limited by the laser line width (ca. 0.4% of the irradiating photon frequency). As noted in previous studies,^27,67^ the employed action spectroscopy technique exhibits a nonlinear dependence of line intensity on transition strength at a given FEL macropulse energy, and thus, the relative intensity of strong or weak lines may be exaggerated or diminished, respectively. Shown in Figure 1b is the IR action spectrum of the unsubstituted complex, [HSO_4_^–^ + H^+^ + HCOO^–^]^−^, acquired between 432 and 2200 cm^–1^. The most intense lines are found at 769 cm^–1^ (b_2_, Figure 1b), 1048 cm^–1^ (b_4_), 1422 cm^–1^ (b_7_), and 1744 cm^–1^ (b_8_). Possible assignments for lines b_2_ and b_4_ include stretching modes of the hydrogen sulfate moiety and displacement modes of the bridging proton, the latter of which are often highly anharmonic and strongly coupled to large-amplitudes modes in AHA^–^ complexes.^13,17,19,27,28^ Line b_7_ is expected to arise from a bridging proton displacement mode, as modes of the conjugate base moieties are not expected in this spectral region. Line b_8_ is readily assigned to a deformation mode of the formate carboxylate moiety and is of particular interest, as previous studies on AHA^–^ homodimers have utilized the location of this band as an indirect reporter of bridging proton location.^26−28^ For the complex studied herein, line b_8_ (1744 cm^–1^) is found near the literature value for the C=O stretching mode of free formic acid (ca. 1770 cm^–1^),^68,69^ indicating that the bridging proton is proximal to the formate moiety, as in structures **1** and **2**.

To better ascertain the vibrational motions associated with each spectral line, IR action spectra of isotopologues of [HSO_4_^–^ + H^+^ + HCOO^–^]^−^ were also acquired, as shown in parts a, c, and d of Figure 1. Substitution of two ^18^O atoms on the formate group yields a red-shift of ca. 30 cm^–1^ of line a_8_ with respect to line b_8_ (Figure 1, parts a and b, respectively), supporting the assignment of this band to a C=O stretching mode of the HCOO^–^···H^+^ group. The position of the remaining spectral lines is largely unchanged (Table S1).

Deuterium substitution of either one or two of the exchangeable hydrogens in the system was also carried out, and the corresponding IR spectra are shown in parts c and d of Figure 1, respectively. The spectral lines c_10_ and d_9_ located at ca. 2155 and 2152 cm^–1^, respectively, are assigned to the O–D stretching mode of the hydrogen sulfate moiety, further indicating a complex resembling structures **1** and **2** rather than the equally shared protons of structure **3**. Comparing the spectrum of the doubly deuterated complex to that of the unsubstituted complex (Figure 1, parts d and b, respectively), lines directly corresponding to b_5_ and b_7_ are not readily identified. In addition, new lines d_5_ and d_6_ appear, and line b_1_ is replaced by the much weaker line d_1_, which may arise from trace presence of the nominally isobaric ^34^S complex or may correspond to a different mode altogether. The modes associated with these bands may involve substantial motion of the bridging proton or deuteron or may result from differences in coupling between these motions and conjugate-base deformation modes. In contrast, lines d_2_, d_3_, d_4_, d_7_, and d_8_ are not substantially shifted from the corresponding lines in the spectrum of the unsubstituted complex (Table S1), indicating the relevant normal modes are largely decoupled from bridging proton or deuteron motion. This result contrasts starkly with the large shifts in low-frequency lines (<1200 cm^–1^) observed upon deuterium substitution in other AHA^–^ complexes such as H_3_O_2_^–^ and the formate proton-bound homodimer.^18,27,37^ It is anticipated that additional intense spectral lines associated with displacement of the bridging proton may be found at photon energies less than 430 cm^–1^.

Finally, the spectrum of the singly deuterated species shown in Figure 3c resembles a combination of the spectra of two distinct isotopomers, one with the deuterium atom localized on the hydrogen sulfate moiety (i.e., DSO_4_^–^···H^+^···^–^OOCH) and one with the deuteron replacing the bridging proton (i.e., HSO_4_^–^···D^+^···^–^OOCH). Deuterium substitution at the hydrogen sulfate moiety is favored energetically, as the decrease in zero-point energy is larger upon substitution of the localized rather than the bridging proton, with an estimated energy difference on the order of 3 kJ mol^–1^. However, with the ions initially held in the ion trap at 298 K, the isotopomer population is likely first equilibrated at this temperature. Depending on the cooling rates and the barrier for interconversion, some ions might be kinetically trapped in the energetically disfavored HSO_4_^–^···D^+^···^–^OOCH form upon cooling to 0.4 K in the helium nanodroplet.^70−73^ The presence of lines attributed to (^−^O_3_SO–D) stretching motion near 2150 cm^–1^ in both spectra (c_10_ and d_9_ in Figure 1, parts c and d, respectively) indicates that at least some ions are in the DSO_4_^–^···H^+^···^–^OOCH form. Further, line positions in the [DSO_4_^–^ + H^+^ + HCOO^–^]^−^/[HSO_4_^–^ + D^+^ + HCOO^–^]^−^ spectrum in Figure 1c are largely unchanged from those of the unsubstituted and doubly deuterated species, respectively, and the spectrum in Figure 1c appears as a combination of those in Figure 1, parts b and d, with likely most singly deuterated ions in the DSO_4_^–^···H^+^···^–^OOCH form.

**Figure 2:**
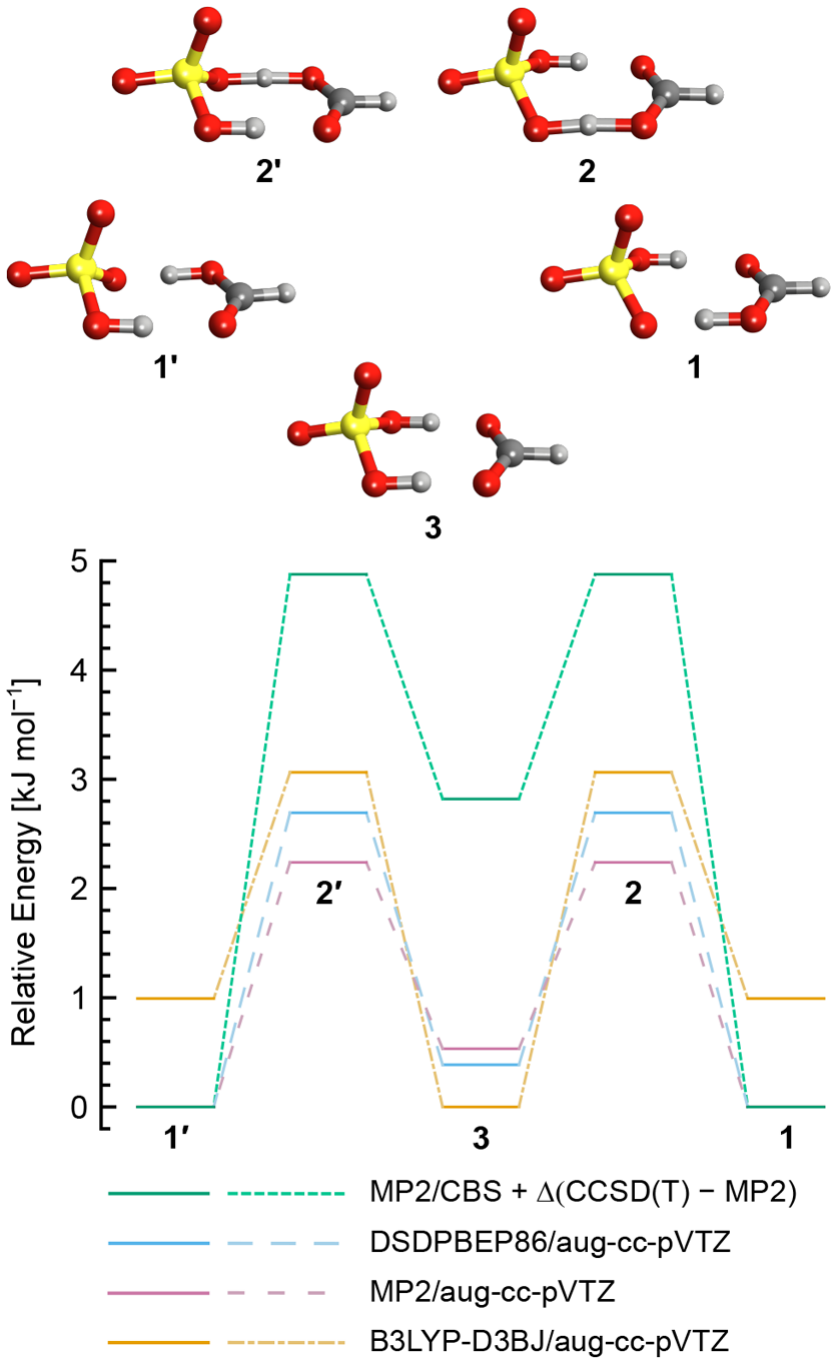
Structures and relative energies of the proton-bound dimer of hydrogen sulfate and formate, [HSO_4_^–^ + H^+^ + HCOO^–^]^−^. Local minima are found in which the proton is localized on either the hydrogen sulfate moiety (**3**) or the formate moiety (**1**). These minima are separated by a transition state featuring a shared proton (**2**). Structures **1′** and **2′** are structurally and energetically equivalent to structures **1** and **2** but are significant in the analysis of the potential energy surface. The magnitude of the proton translocation barrier and relative energies are strongly dependent on the level of theory employed.

The experimental IR spectra are consistent with a structure of the proton-bound heterodimer of hydrogen sulfate and formate that resembles **1** or **2**, shown schematically in Scheme 1. Note that the population of a structure resembling **2** is only feasible if the zero-point energy for bridging proton motion exceeds the barrier height, resulting in significant delocalization of the bridging proton. To further assess the form observed experimentally, these structures were also investigated using electronic structure methods. Shown in Figure 2 are the computed structures and corresponding relative energies for the identified local minima and transition states of this system. As discussed in the introduction, structure **3** features a sulfuric acid molecule complexed with a formate anion, H_2_SO_4_(HCOO^–^), and belongs to the *C*_*s*_ point group. Structure **1**, in contrast, comprises a hydrogen sulfate anion complexed with a formic acid molecule, HSO_4_^–^(HCOOH), and belongs to the *C*_1_ point group. These two minima are connected by a transition state (structure **2**) in which the proton is shared between the hydrogen sulfate and formate moieties. Because either proton in structure **3** can be transferred from the sulfuric acid moiety to the formate residue, there exist two equivalent transition states and minima on the potential energy surface, as denoted in Figure 2 by structures **2**, **2′** and **1**, **1′**, respectively.

Examining the relative energies of the low-energy structures, a notable discrepancy is observed between the results from B3LYP-D3BJ, a hybrid density functional combined with an empirical correction for dispersion interactions,^47−49^ and the results from MP2 and DSDPBEP86, both of which incorporate a second-order correction to account for correlation energy.^51−53^ Whereas the B3LYP-D3BJ method predicts structure **3** to be lowest in energy, the MP2 and DSDPBEP86 methods find that structure **1** is lowest in energy. All three approaches significantly underestimate the stability of structure **1** compared to MP2 calculations employing a complete basis set (CBS) extrapolation^54^ and a correlation energy correction for the difference between CCSD(T) and MP2.^61^ As a result, the transition-state energy of **2** is also underestimated by all methods in comparison to the MP2/CBS benchmark. Because the magnitude of this barrier is on the order of the anticipated zero-point energy for proton translocation, the underestimation by more affordable electronic structure methods such as hybrid DFT represents a significant challenge to the assessment of proton delocalization, as such low-cost methods are typically employed for more computationally intensive approaches such as path-integral molecular dynamics.^74−77^

**Figure 3:**
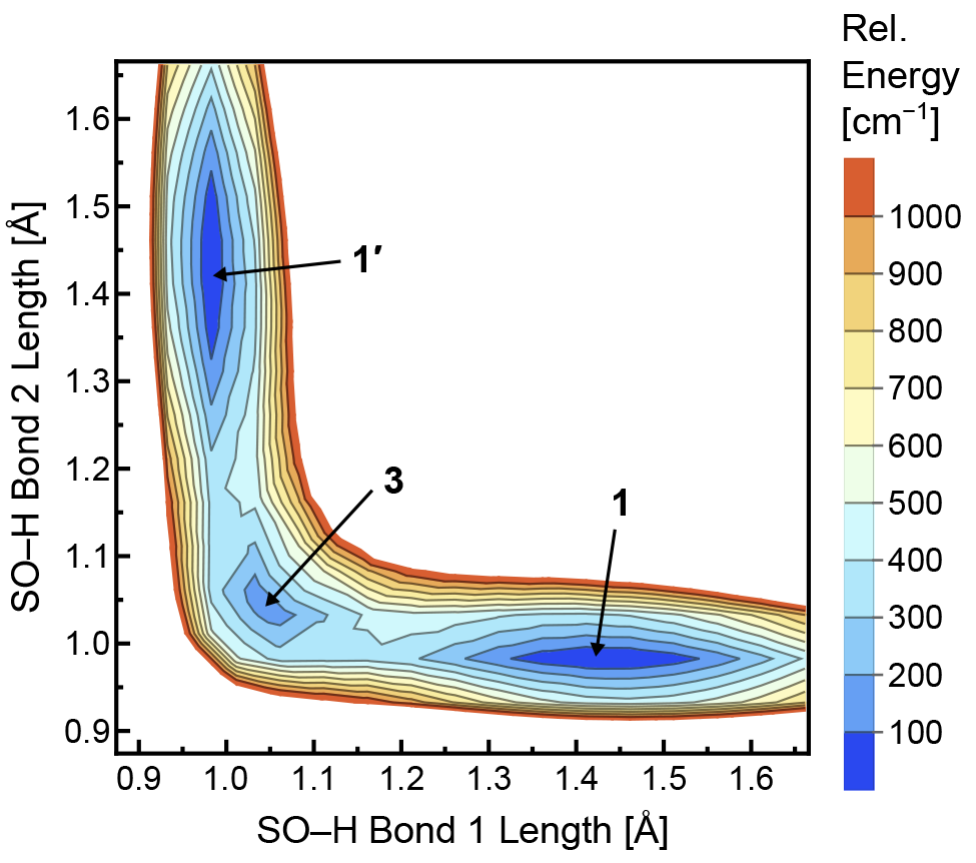
Relaxed potential energy surface (PES) of [HSO_4_^–^ + H^+^ + HCOO^–^]^−^ as a function of fixed SO–H bond lengths. The on-diagonal minimum corresponds to the symmetric structure **3**, whereas the off-diagonal minima correspond to the structures **1** and **1′**, with the transition-state structures **2** and **2′** located along the lowest-energy path between the minima. When one proton is localized to yield hydrogen sulfate (SO–H bond length of 0.983 Å), the energetic penalty associated with changes in the length of the remaining SO–H bond is relatively small (<350 cm^–1^).

To further investigate the properties of the [HSO_4_^–^ + H^+^ + HCOO^–^]^−^ system, a relaxed potential energy surface (PES) was constructed by fixing the SO–H bond lengths while optimizing all other geometric parameters at the DSDPBEP86/aug-cc-pVTZ level of theory. Single-point energies for each optimized structure were subsequently calculated at the CCSD(T)/aug-cc-pVTZ level of theory. The resulting PES is shown in Figure 3. The local minimum on the diagonal corresponds to the symmetric structure **3**, whereas the two off-diagonal minima correspond to the asymmetric structures **1** and **1′**. The transition states **2** or **2′** are then found on the lowest-energy path between the off-diagonal and on-diagonal minima, although the grid spacing of 0.05 Å is not sufficiently small to fully capture the low-energy pathway. Within each off-diagonal potential well, there is a strong energetic penalty for displacement of the proton localized at the hydrogen sulfate moiety from its minimum-energy length of 0.983 Å. In contrast, the potential governing displacement of the remaining SO–H bond, which corresponds to the distance between hydrogen sulfate and the bridging proton, is comparatively shallow, with displacement from the minimum-energy bond length of 1.411 Å to 1.25 or 1.55 Å incurring an energetic penalty of less than 300 cm^–1^. Note that, in this relaxed PES, changes in the SO–H bond length do not necessarily represent changes in the corresponding COO–H bond length. For displacement of a single SO–H bond while holding the remaining SO–H bond at 0.983 Å, the region beyond 1.35 Å is dominated by an increase in the distance between the two conjugate base moieties rather than a concomitant decrease in the COO–H bond length. In contrast, between 1.1 and 1.3 Å, the conjugate base separation is nearly constant at ca. 2.4 Å, and any increase in SO–H bond length is accompanied by a decrease in COO–H bond length.

The PES shown in Figure 3 reveals several noteworthy properties of the [HSO_4_^–^ + H^+^ + HCOO^–^]^−^ system. Within the potential wells corresponding to structures **1** or **1′**, there is little energetic penalty for displacement of the bridging proton from its minimum-energy position at the formate residue. As noted in the previous discussion, this displacement is largely uncorrelated with motion of the proton on hydrogen sulfate until after the transition-state crossing into the on-diagonal potential well representing structure **3**. As a result of this shallow potential, NQEs related to the zero-point energy can be expected to significantly increase the displacement of the proton from the energy minimum, effectively elongating the COO–H bond. The extent of this elongation or proton delocalization is strongly dependent on the relative energy of the transition state **2** or **2′**, which, as discussed previously, is difficult to predict accurately with affordable electronic structure methods. At the highest level of theory employed in this work, MP2/CBS+Δ(CCSD(T)-MP2), the barrier height was calculated to be 4.88 kJ mol^–1^ (408 cm^–1^), which is expected to be on the order of the zero-point energy for proton translocation. The structural impact of a zero-point energy above the barrier is difficult to predict, as such a result may lead to correlation between the behavior of both bridging protons due to the symmetry of the PES.

**Figure 4:**
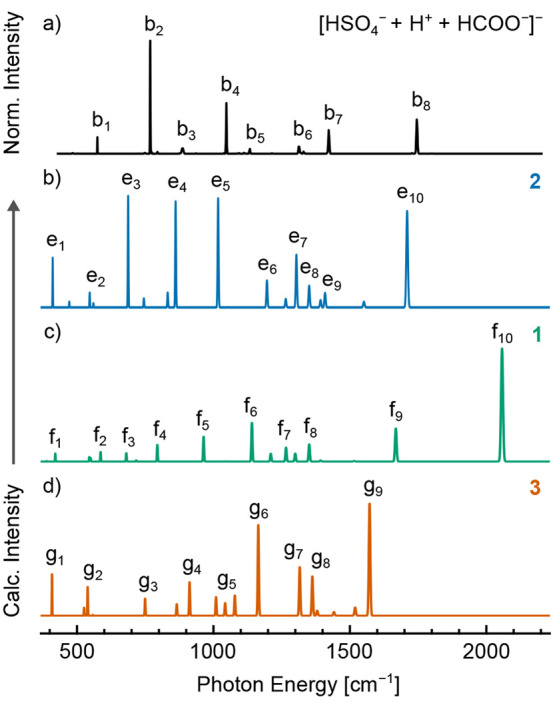
Experimental IR action spectrum of [HSO_4_^–^ + H^+^ + HCOO^–^]^−^ captured in helium nanodroplets (a) and predicted harmonic IR spectra of structures **2** (b), **1** (c), and **3** (d) computed at the DSDPBEP86/aug-cc-pVTZ level of theory and scaled by a factor of 0.985. Better qualitative agreement with experiment is observed for structures **1** and **2** than for structure **3**.

The expected proton delocalization resulting from NQEs and the anharmonicity of vibrational modes involving proton displacement represent significant hindrances to structural assignment from computed harmonic IR spectra. As shown in Figure 4, the experimental IR spectrum of the unsubstituted complex, [HSO_4_^–^ + H^+^ + HCOO^–^]^−^ (Figure 4a), does not exhibit good agreement with the computed harmonic IR spectra of structures **2**, **1**, and **3** (parts b–d of Figure 4, respectively) calculated at the DSDPBEP86/aug-cc-pVTZ level of theory. Interestingly, the IR spectrum of the transition state structure **2** (Figure 4b) exhibits qualitative agreement with the experimental spectrum (Figure 4a), most notably in the position of the C=O stretching mode (line e_10_ vs line b_8_). In general, the computed spectra of structures **1** and **2** exhibit better agreement with experiment than the spectrum of structure **3**. These trends are also observed for the spectra of the doubly deuterated complex (Figure S1) and are consistent with the assignment of a structure resembling **1** or **2** based on the analysis of experimental IR action spectra. However, the likely significant vibrational anharmonicities and strong coupling between bridging proton translocation and conjugate base deformation^17,18,27,28^ render harmonic IR spectra unreliable for structural assignment. Addressing such issues requires a more comprehensive mapping of the PES^78,79^ or alternate methods to address NQEs, such as path integral molecular dynamics.^21,75−77,80,81^

The analysis presented herein has not addressed the contribution of anion interactions with the surrounding helium to the observed line positions. Although such interactions are generally expected to cause minimal perturbation,^40,80−82^ recent spectroscopic interrogations of helium-solvated cations have observed shifts of up to 50 cm^–1^ with respect to bare cations.^83,84^ The situation for anionic species remains much more poorly known. However, theoretical investigations suggest that the solvation shell around anions remains liquid-like,^85,86^ potentially decreasing spectral perturbation. Previous results comparing spectra of messenger-tagged anions to those of anions in helium nanodroplets did not find evidence for large shifts,^27,39^ although both methods likely produce modest shifts with respect to bare ions. The specialized electronic structure methods recently developed by Marx and co-workers have been employed to investigate the behavior of cationic species in superfluid helium and may also yield new insight for anionic complexes.^74,80,81,87,88^ Further experimental and theoretical investigations are needed to better understand the behavior of anionic species in helium nanodroplets.

## Conclusion

The proton-bound dimer of formate and hydrogen sulfate features a shallow potential energy surface for bridging proton translocation, impeding unambiguous structural assignment. Experimental characterization of [HSO_4_^–^ + H^+^ + HCOO^–^]^−^ and its isotopologues by IR action spectroscopy of ions trapped in helium nanodroplets provides evidence for a structure resembling **1** or **2**, in which a single bridging proton is displaced from the hydrogen sulfate moiety. The extent to which this proton is shared or localized at the formate residue, however, is difficult to ascertain. Further assessment of this structure employing more advanced computational approaches that address NQEs is necessary to confirm the structure observed experimentally. This system serves as a reminder of the importance of accounting for NQEs when addressing the structure of ionic hydrogen bonding complexes.

## Supporting Information Available

Additional computed spectra of isotopologues, experimental and computational spectral line positions, and calculated energies (PDF)
Tabulated experimental spectra and xyz coordinates and normal modes for theoretical structures (TXT).
