# Supplementary material for: Helium Nanodroplet Infrared Action Spectroscopy of the Proton-Bound Dimer of Hydrogen Sulfate and Formate: Examining Nuclear Quantum Effects
Source: J Phys Chem A. 2021 Oct 15;125(42):9279–87. doi: 10.1021/acs.jpca.1c05705 (PMC8558860; doi:10.1021/acs.jpca.1c05705)
Supplement: Supplementary file 1 — jp1c05705_si_001.pdf [file jp1c05705_si_001.pdf]

Supporting Information: Helium Nanodroplet Infrared Action Spectroscopy of the Proton-Bound Dimer of Hydrogen Sulfate and Formate: Examining Nuclear Quantum Effects

Daniel A. Thomas[†]*, Martín Taccone, Katja Ober, Eike Mucha, Gerard Meijer, and Gert von Helden**

Fritz-Haber-Institut der Max-Planck-Gesellschaft, Faradayweg 4–6, 14195 Berlin, Germany

Corresponding Author

*Email: helden@fhi-berlin.mpg.de

Table of Contents

Computed Infrared Spectra for Isotopologue Structures S2

Tabulation of Experimental and Computed Spectral Line Positions S3

Computed Energies of Structures 1, 2, and 3 S5

Computed Infrared Spectra for Isotopologue Structures

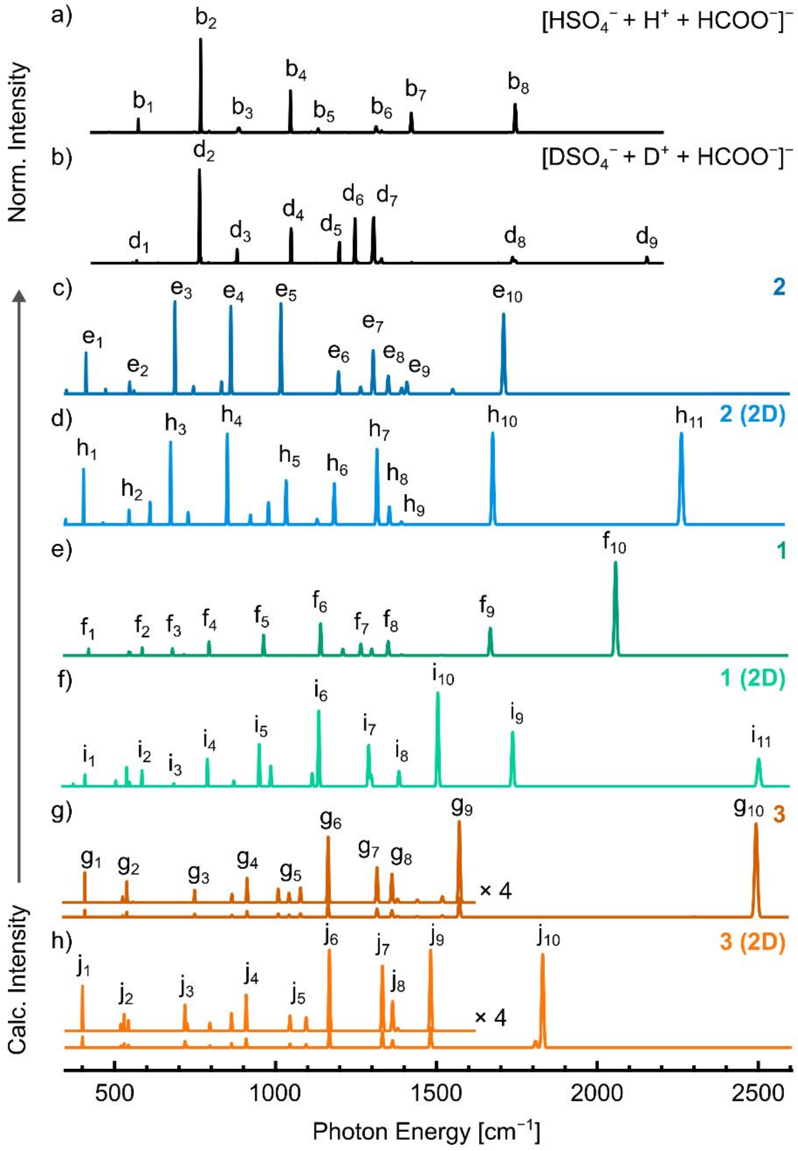

Figure S1. Experimental IR action spectrum of (a) [HSO_4^-+H^++$HCOO^-$]$^-$ and (b) [DSO_4^-+D^++$HCOO^-$]$^-$ captured in helium nanodroplets and predicted harmonic IR spectra of structures **2** (c), **1** (e), and **3** (g) and the corresponding doubly deuterated (2D) isotopologues **2**

(2D) (d), **1** (2D) (f), and **3** (2D) (h). Predicted harmonic IR spectra were calculated at the DSDPBEP86/aug-cc-pVTZ level of theory and scaled by a factor of 0.985.

Tabulation of Experimental and Computed Spectral Line Positions

Table S1. Tabulation of Selected Experimental Spectral Lines for $[HSO_4^{-}+H^{+}+HCOO^{-}]^{-}$ and isotopologues.[a]

Line	Freq. [cm^{-1}]	Line	Freq. [cm^{-1}]	Line	Freq. [cm^{-1}]	Line	Freq. [cm^{-1}]
b_1	576	a_1	576	c_1	576	d_1	571
b_2	769	a_2	769	c_2	768	d_2	766
b_3	888	a_3	883	c_3	884	d_3	883
b_4	1048	a_4	1048	c_4	1049	d_4	1050
b_5	1134	a_5	1133	c_5	1211	d_5	1200
				c_6	1255	d_6	1248
b_6	1314	a_6	1314	c_7	1314	d_7	1306

**b_7**	1412	**a_7**	1422	**c_8**	1422		
**b_8**	1744	**a_8**	1717	**c_9**	1745	**d_8**	1736
				**c_{10}**	2155	**d_9**	2152

a. Spectral line labels correspond to those given in Figure 2 and Figure 3.

Table S2. Tabulation of Selected Computed Spectral Lines for $[HSO_4^{-}+H^{+}+HCOO^{-}]^{-}$ and $[DSO_4^{-}+D^{+}+HCOO^{-}]^{-}$.[a]

Line	Freq. [cm^{-1}]	Line	Freq. [cm^{-1}]	Line	Freq. [cm^{-1}]	Line	Freq. [cm^{-1}]	Line	Freq. [cm^{-1}]	Line	Freq. [cm^{-1}]
**e_1**	413	**h_1**	404	**f_1**	422	**i_1**	411	**g_1**	411	**j_1**	404
**e_2**	548	**h_2**	546	**f_2**	588	**i_2**	587	**g_2**	541	**j_2**	522, 533, 546
**e_3**	689	**h_3**	675	**f_3**	682	**i_3**	686	**g_3**	751	**j_3**	721
**e_4**	862	**h_4**	851	**f_4**	796	**i_4**	790	**g_4**	913	**j_4**	911
**e_5**	1018	**h_5**	1033	**f_5**	965	**i_5**	951	**g_5**	1010, 1043,	**j_5**	1046, 1096

									1079		
e_6	1196	h_6	1183	f_6	1141	i_6	1136	g_6	1165	j_6	1168
e_7	1304	h_7	1316	f_7	1266	i_7	1290	g_7	1316	j_7	1333
e_8	1351	h_8	1354	f_8	1351	i_8	1384	g_8	1362	j_8	1364
e_9	1409	h_9	1391	f_9	1667	i_9	1737	g_9	1571	j_9	1482
e_{10}	1709	h_{10}	1675	f_{10}	2056	i_{10}	1505	g_{10}	2490	j_{10}	1829
		h_{11}	2261			i_{11}	2499				

a. Spectral line labels correspond to those given in Figure 2 and Figure S2.

Computed Energies of Structures 1, 2, and 3

Table S3. Computed Energies of $[HSO_4^{-}+H^{+}+HCOO^{-}]^{-}$ Structures 1, 2, and 3.[a]

Struct. Num.	Energy[b] MP2/ aug-cc-pVTZ	Zero-Point Energy[b] MP2/ aug-cc-pVTZ	Energy[b] MP2/ aug-cc-pVQZ	Energy[b] MP2/ aug-cc-pV5Z	Energy[b] CCSD(T)/ aug-cc-pVTZ	Energy[b] CBS + CCSD(T) Correct	Energy[c] DSDPBEP86/aug-cc-pVTZ	Zero-Point Energy[c] DSDPBEP86/ aug-cc-pVTZ	Energy[d] B3LYP-D3BJ/ aug-cc-pVTZ	Zero-Point Energy[d] B3LYP-D3BJ/ aug-cc-pVTZ
1	−888.44162	0.06220	−888.61400	−888.68507	−888.51438	−888.80768	−888.74762	0.06228	−889.80755	0.06130
2	−888.44076	0.05919	−888.61304	−888.68398	−888.51295	−888.80583	−888.74660	0.05900	−889.80676	0.05810
3	−888.44141	0.06123	−888.61366	−888.68459	−888.51377	−888.80661	−888.74748	0.06129	−889.80793	0.06050

a. Values reported in Hartrees.

b. Geometry optimization at the MP2/aug-cc-pVTZ level of theory.

c. Geometry optimization at the DSDPBEP86/aug-cc-pVTZ level of theory.

d. Geometry optimization at the B3LYP-D3BJ/aug-cc-pVTZ level of theory.
